# Blood Glucose and Risk of Incident and Fatal Cancer in the Metabolic Syndrome and Cancer Project (Me-Can): Analysis of Six Prospective Cohorts

**DOI:** 10.1371/journal.pmed.1000201

**Published:** 2009-12-22

**Authors:** Tanja Stocks, Kilian Rapp, Tone Bjørge, Jonas Manjer, Hanno Ulmer, Randi Selmer, Annekatrin Lukanova, Dorthe Johansen, Hans Concin, Steinar Tretli, Göran Hallmans, Håkan Jonsson, Pär Stattin

**Affiliations:** 1 Department of Surgical and Perioperative sciences, Urology and Andrology, Umeå University, Umeå, Sweden; 2 Institute of Epidemiology, Ulm University, Ulm, Germany; 3 Department of Public Health and Primary Health Care, University of Bergen, Bergen, Norway; 4 Norwegian Institute of Public Health, Bergen, Norway; 5 Department of Surgery, Malmö University Hospital, Malmö, Sweden; 6 Department of Medical Statistics, Informatics and Health Economics, Innsbruck Medical University, Innsbruck, Austria; 7 Division of Epidemiology, Norwegian Institute of Public Health, Oslo, Norway; 8 Division of Cancer Epidemiology, German Cancer Research Center, Heidelberg, Germany; 9 Agency for Preventive and Social Medicine, Bregenz, Austria; 10 Institute of Population-based Cancer Research, The Cancer Registry of Norway, Oslo, Norway; 11 Department of Public Health and Clinical Medicine, Nutritional Research, Umeå University, Umeå, Sweden; 12 Department of Radiation Sciences, Oncology, Umeå University, Umeå, Sweden; University of Cambridge Institute of Public Health, United Kingdom

## Abstract

**Background:**

Prospective studies have indicated that elevated blood glucose levels may be linked with increased cancer risk, but the strength of the association is unclear. We examined the association between blood glucose and cancer risk in a prospective study of six European cohorts.

**Methods and Findings:**

The Metabolic syndrome and Cancer project (Me-Can) includes cohorts from Norway, Austria, and Sweden; the current study included 274,126 men and 275,818 women. Mean age at baseline was 44.8 years and mean follow-up time was 10.4 years. Excluding the first year of follow-up, 18,621 men and 11,664 women were diagnosed with cancer, and 6,973 men and 3,088 women died of cancer. We used Cox regression models to calculate relative risk (RR) for glucose levels, and included adjustment for body mass index (BMI) and smoking status in the analyses. RRs were corrected for regression dilution ratio of glucose. RR (95% confidence interval) per 1 mmol/l increment of glucose for overall incident cancer was 1.05 (1.01–1.10) in men and 1.11 (1.05–1.16) in women, and corresponding RRs for fatal cancer were 1.15 (1.07–1.22) and 1.21 (1.11–1.33), respectively. Significant increases in risk among men were found for incident and fatal cancer of the liver, gallbladder, and respiratory tract, for incident thyroid cancer and multiple myeloma, and for fatal rectal cancer. In women, significant associations were found for incident and fatal cancer of the pancreas, for incident urinary bladder cancer, and for fatal cancer of the uterine corpus, cervix uteri, and stomach.

**Conclusions:**

Data from our study indicate that abnormal glucose metabolism, independent of BMI, is associated with an increased risk of cancer overall and at several cancer sites. Our data showed stronger associations among women than among men, and for fatal cancer compared to incident cancer.

*Please see later in the article for the Editors' Summary*

## Abstract 2

Tanja Stocks and colleagues carry out an analysis of six European cohorts and confirm that abnormal glucose metabolism is linked with increased risk of cancer overall and at specific sites.

## Editors' Summary

**Background:**

Large prospective population-based research studies can have the power to discover new associations, and to verify previously proposed associations, between specific risk factors and the subsequent occurrence of disease. One such study, the “Me-Can” (Metabolic syndrome and Cancer project) is investigating associations between cancer incidence and a cluster of metabolic risk factors that make up metabolic syndrome: a large waistline; a high level of fats called triglycerides in the blood; a low level of “good” cholesterol; high blood pressure; and raised blood glucose (hyperglycemia). Here the researchers investigate the associations between one of these risk factors—raised blood glucose—and cancer. It is normal for blood glucose levels to vary before and after meals, but raised levels that persist long-term are known to lead to organ damage and severe complications. It is thought that more than 30% of cancer-related deaths could be prevented by modifying key risk factors, such as tobacco control, modifying diet, staying active, and limiting exposure to environmental risk factors.

**Why Was This Study Done?:**

A previous large research study (including roughly 1.3 million men and women, conducted in Korea) has already evaluated the association between high blood glucose levels and cancer risk, and found that high blood glucose levels were linked with increased risk of cancer—both getting it and dying from it. Studies in European and US populations have also found a link, but they considered relatively small numbers of people and so these could not be used to calculate the risk with respect to specific cancer sites. The researchers carrying out the Me-Can project wanted to verify whether the associations reported in the Korean study also held true for European populations.

**What Did the Researchers Do and Find?:**

The researchers identified 274,126 men and 275,818 women from existing health studies in Norway, Austria, and Sweden for whom data had been recorded on blood glucose level, height, and weight. For each participant a baseline measurement was defined, consisting of data from the first health examination, which had complete data (including a blood glucose measurement and whether the participant smoked). The participants were tracked via national registers for up to around 25 years after the baseline measurement but most commonly for around a decade. Any cancer diagnosis was recorded, whether the participant survived to the end of the study, and causes of death for participants who died during the study. The researchers analyzed the data to assess whether a higher blood glucose level was associated with increased risk of certain cancers, in both men and women. The researchers took weight for height, and smoking into account and adjusted for measurement error from additional blood glucose measurements. The researchers found that, overall, the higher the level of blood glucose, the higher the risk of getting and dying from cancer. Average normal blood glucose levels are about 5 mmol/l, also expressed as 5 mM or 90 mg/dl. For each additional 1 mmol/l increase in blood glucose level, the risk of getting cancer was increased by 5% for men and 11% for women.

**What Do These Findings Mean?:**

The authors concluded that high blood glucose is associated with increased cancer risk. The results largely confirm findings from the Korean study, although there are some differences in the risks of cancers at some specific sites, which may be due to differences in the populations such as genetics, diet, and rates of smoking. Among the strengths of the study are its large sample size and that glucose were measured more than once for many individuals in the study. However, the study is limited in that the researchers did not have data on other possible factors such as genetics, physical activity, or dietary factors, which are linked to cancer incidence and also may be related to blood glucose levels. The researchers propose that controlling blood glucose may lower cancer risk in the population. Although this interpretation is consistent with the data, the study design cannot conclusively demonstrate a causal association between glucose levels and cancer risk.

**Additional Information:**

Please access these Web sites via the online version of this summary at http://dx.doi.org/10.1371/journal.pmed.1000201.

- The US National Cancer Institute provides online information and statistics on cancer, including risk factors for cancer
- The American Heart Association provides information on sugars in the diet, including helpful hints on how to reduce the amount eaten
- The UK's National Health Service's Change4Life campaign provides information and ideas for those wishing to make their lifestyle, including diet, more healthy
- Cancer Research UK is the world's leading charity dedicated to beating cancer through research. Its websites provide information about cancer and the research it funds

## Introduction

Elevated blood glucose has been associated with an increased risk of cancer overall in several prospective studies [1]–[6]. The strongest evidence comes from a Korean cohort study of 1.3 million men and women that reported an increased risk of incident as well as of fatal cancer in individuals with high glucose levels [1]. Prospective studies of glucose and cancer risk in cohorts of European and US populations have been much smaller, and these studies did not concurrently report on risk of incident and fatal cancer [2]–[7]. Previous results from cohorts in Austria [2] and Sweden [3] included in the current study, also indicated that elevated fasting glucose is related to an increased risk of overall incident cancer. However, the relatively modest sample size in these studies resulted in limited power to estimate risks for individual cancer sites. Furthermore, exposure assessment by glucose measurement at a single occasion entails a substantial random error owing to technical measurement error and within-person variation of blood glucose level [8],[9]. Such inaccuracy of exposure assessment will dilute the association with outcome, i.e., regression dilution bias [8],[10],[11]. In several prospective studies of metabolic factors and risk of cardiovascular disease, data from multiple examinations have been used to correct risk estimates for random error in exposure classification, which resulted in substantially stronger associations than estimates on the basis of uncorrected exposures [12]–[14]. To date, correction for random error has only been performed in one study on glucose and cancer risk [3].

The aim of this study was to investigate the association between blood glucose and risk of incident and fatal cancer overall and at specific sites, as well as all-cause mortality, in a large study of six European cohorts including correction for random error in glucose levels.

## Material and Methods

### Me-Can

The Metabolic syndrome and Cancer project (Me-Can) includes data from population-based cohorts in Norway, Austria, and Sweden. A detailed description of Me-Can has recently been published [15]. In brief, the Norwegian cohorts includes the Oslo study I cohort (Oslo) [16],[17], the Norwegian Counties Study (NCS) [18],[19], the Cohort of Norway (CONOR) [20], and the Age 40-programme (40-y) [21]. The Austrian cohort consists of the Vorarlberg Health Monitoring and Prevention Programme (VHM&PP) [2], and the Swedish cohorts are the Västerbotten Intervention Project (VIP) [22], and the Malmö Preventive Project (MPP) [23],[24]. Written informed consent was obtained from all participants included in this study, and the study was approved by research ethical committees in the respective countries.

Data on height, weight, blood pressure, and blood, plasma, or serum levels of glucose, total cholesterol, and triglycerides had been collected at health examinations in all cohorts. Height and weight were measured in a similar way in all cohorts; without shoes and with light indoor clothing. In the Norwegian cohorts, fasting was not required before the examination, and fasting time was recorded as <1 h, 1–2, 2–4, 4–8, or >8 h. Fasting time in the VIP was recorded as <4 h, 4–8, or >8 h, and from 1992, participants were asked to fast for at least 8 h before the examination. In the MPP and after the initial 3 y in the VHM&PP, a minimum of 8 h fasting time before blood draw was implemented. Glucose levels were measured in the Oslo and the NCS in serum glucose with a nonenzymatic method; in CONOR and the 40-y cohort, serum/enzymatic; in the VHM&PP and the VIP, plasma/enzymatic; and in the MPP, whole blood/enzymatic. In the Norwegian cohorts, the nonenzymatic method used during the first study period yielded 0.8–1.1 mmol/l higher levels than by the use of an enzymatic method [25]. Data from several health examinations were available for a subset of individuals in some of the Me-Can cohorts [15], and for each person in the study, data from one health examination constituted the baseline observation, described as follows.

### Follow-up and Selection of Participants

Each of the cohorts was linked to the respective national registers for identification of (a) cancer diagnosis, (b) migration, (c) vital status, and (d) cause of death, with death attributed to cancer if the underlying cause of death was cancer. Follow-up for each of the cohorts includes the year as follows: Norwegian cohorts, (a–c) 2005, (d) 2004; the VHM&PP, (a) 2003, (b) no information available, (c, d) 2003; the VIP and the MPP (a–c) 2006, (d) 2004.

**Figure 1:**
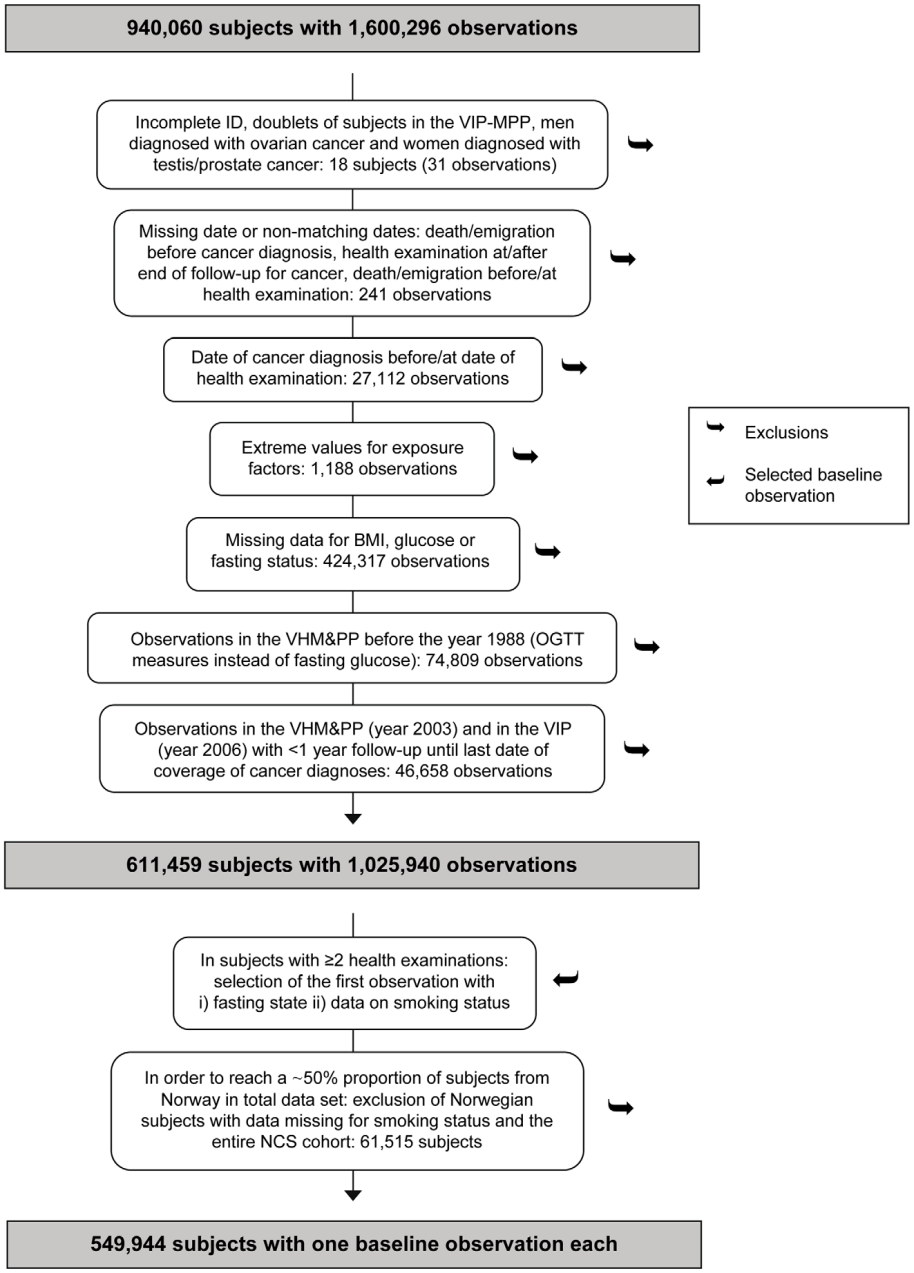
Flowchart of data cleaning and selection of individuals/observations. OGTT, oral glucose tolerance test. NCS, Norwegian Counties Study.

Selection of individuals for the study is described in Figure 1. From the original data with 904,060 individuals and 1,600,296 observations, we excluded observations with: nonmatching data, a cancer diagnosis at or before the date of health examination, extreme values of metabolic factors [15] (<1 mmol/l for glucose and <15 or >60 kg/m^2^ for body mass index [BMI]), missing data for BMI, glucose or fasting time, a shorter time than 1 y between the date of examination and end of follow-up for cancer incidence, and observations in the VHM&PP that included data on postload glucose instead of fasting glucose. Out of the 574,356 excluded observations, 414,629 observations were excluded in the Norwegian cohorts in individuals for whom data on glucose were missing, as blood glucose had not been measured as a standard in these cohorts throughout all time-periods. From the remaining 611,459 individuals with 1,025,940 observations, we selected the first observation for each individual, and if data from a fasting state and data on smoking status were available, the first of these observations was selected. Thus, for each individual, data were included from the first health examination with complete data to comprise the baseline set of measurements. Due to policy restrictions imposed by the Norwegian Institute of Public Health that the proportion of Norwegian individuals in Me-Can studies should not exceed approximately 50% (56% after the above selection), we further excluded 1,868 individuals in Norway without data on smoking status, and the entire NCS cohort (*n* = 59,647). The reason for excluding an entire cohort was to keep the included Norwegian cohorts intact and to keep down the number of strata in statistical analyses, as a large number of strata reduce statistical power. We excluded the NCS cohort as it consisted of approximately the number of individuals that was required to be excluded. The final dataset included 549,944 individuals, 274,126 men and 275,818 women.

### Categorisation of Cancers

Incident and fatal cancers, categorised according to the International Classification of Diseases, seventh revision (ICD-7) codes, were grouped into cancer sites as grouped in the Eurostat European shortlist for cause of death [26], which was used for cause of death classification in the Norwegian cohorts. Incident cancers were further divided into relevant subgroups. Relative risks (RR) for incident and fatal cancer at specific sites are presented separately for men and women if the number of cases in each group was >50, and risks are presented for men and women combined if the number of cases in each group was ≤50 and if the total number of cases was >80.

### Statistical Analysis

In order to reduce the probability of reverse causation, rates, RRs and absolute risks were calculated with follow-up starting 1 y after the baseline examination. Individuals were followed until the date of event, i.e., cancer diagnosis or cancer death, or until the date of death from any cause, emigration, or end of follow-up, whichever occurred first. Rates were directly age-standardized in 5-y categories, using the European standard population as the reference [27]. We used Cox proportional hazards regression to calculate hazard ratios, denoted as RRs, for glucose levels with risk of incident and fatal cancer, and of death from all causes. Age was used as time variable and all estimates were stratified by subcohort, sex, and by categories of birth date: before 1923, 1923–1930, 1931–1938, 1939–1946, 1947–1954, 1955, and later. We estimated RR for glucose levels in quintiles and deciles, for which cut-off levels were calculated within each subcohort, sex, and category of fasting time. *p* for trend over quintiles and deciles refers to the *p*-value for the Wald test of a linear risk estimate, assigning participants included in each analysis the mean sex- and cohort-specific glucose level within the corresponding quantile. RR was also assessed for glucose as a continuous variable, i.e., per 1 mmol/l increment. In order to exclude outliers, these analyses were restricted to individuals with glucose levels lower than 10 mmol/l (99% of individuals). All analyses included adjustment for age at measurement (continuous), BMI (categories: <22.5, 22.5 to <25.0, 25.0 to <27.5, 27.5 to <30.0, 30.0 to <32.5 kg/m^2^) and smoking status (categories: never smoker, ex-smoker, current smoker, and unknown), and analyses of glucose as a continuous variable were also adjusted for fasting time.

We calculated regression dilution ratio (RDR) of glucose in order to adjust RRs for random error in glucose level [8],[10],[11]. RDR was calculated on the basis of data from repeated health examinations in 133,820 individuals, including 406,364 observations, in the full Me-Can cohort. Only repeated measurements with the same fasting time and in the same cohort as at baseline, and with data on smoking status, were used. However, as the same method for glucose measurement had been used in the Oslo and the NCS cohorts, and in the CONOR and 40-y cohorts, participants with measurements in the Oslo and in the NCS, or in CONOR and in the 40-y cohort, were included in analyses. Mean time between the baseline measurement and repeated measurements was 6.9 y (standard deviation [SD] = 3.9). We used a linear mixed effects model, similar to that described by Wood et al. [11], which included age at baseline, fasting time, smoking status, sex, and time from baseline as fixed effects, and cohort as random effect. RDR was estimated separately for men and women, and combined, in models for (a) glucose standardised within cohort, sex and fasting time, and for (b) glucose only including individuals with a baseline glucose level lower than 10 mmol/l. Model (a) was used to predict RDR among individuals in the current study with data on smoking status, for correction of RRs in quantiles, and model (b) was used to predict RDR among individuals with data on smoking status and with a glucose level lower than 10 mmol/l, for correction of RRs of per 1 mmol/l increment. RDR was predicted for the time point at 5 y after baseline measurement, i.e., half the follow-up time [8],[10],[11]. We used the mean of predicted RDRs for correction of RR, which resulted in RDRs for quantile analyses of: 0.30 among men, 0.30 among women, and 0.31 overall, and in analyses of per 1 mmol/l increment: 0.40 among men, 0.43 among women, and 0.41 overall. Correction of RRs for RDR was obtained by exp(log(RR)/RDR), using the sex-specific RDR in analyses that included men or women only, and using the combined RDR in analyses that included both sexes.

Cox proportional hazards assumption was checked for glucose and covariates by the statistical test of Schoenfeld residuals. For some cancers, there was an indication of violation of proportionality for BMI or smoking status, but as RRs were very similar with and without stratification of the variable within the model, BMI and smoking status were not kept as stratum in the final model. For a few cancers there was an indication of violation of the proportionality over age for glucose; however, we report RRs only in the full study group and not in subgroups of age. Interaction between glucose and (a) BMI, (b) fasting time, and (c) cohort on the risk of overall incident and fatal cancer was checked by analysing RRs in subgroups of BMI, fasting time, and cohort, and by performing likelihood ratio tests comparing the model used to assess RR per 1 mmol/l increment with a model that additionally included a product term of continuous glucose and categories of BMI, fasting time, or cohort, respectively. Interaction between glucose and fasting time was assessed in the Norwegian cohorts. Evidence of a nonlinear association between glucose and risk of overall incident and fatal cancer was tested by likelihood ratio test, comparing the model with glucose as a continuous variable with a model that also included an x^2^ term of glucose. In order to assess linearity across the whole glucose range, all individuals were included in this analysis. Absolute risks of incident and fatal cancer between 50 and 70 y of age were calculated as described by Gail et al. [28]. For this method, risk of cancer and of dying from other causes than cancer was derived from the cohort for ages 50 to 60 y and 60 to 70 y, respectively. Statistical analyses were performed in Stata (version 9.2, StataCorp LP), and R (version 2.7.2, used for RDR calculation).

## Results

### Baseline Characteristics and Follow-up

Mean age at baseline was 44.7 y (SD = 11.6) in men and 45.0 y (SD = 12.8) in women (Table 1). The prevalence of overweight or obesity, i.e., BMI 25 kg/m^2^ or higher, was 56% among men and 42% among women. All participants in the VHM&PP and the MPP and 90% of participants in the VIP had fasted >8 h before the health examination, whereas 95% of participants in the Norwegian cohorts had fasted <8 h. Among individuals that had fasted >8 h, 8% of men and 6% of women had impaired glucose levels according to the World Health Organization definition [29] (6.1–6.9 mmol/l in serum/plasma or 5.6–6.0 mmol/l in whole blood), and 4% of men and 3% of women had diabetic glucose levels (≥7.0 mmol/l in serum/plasma or ≥6.1 mmol/l in whole blood). Baseline age and BMI increased for each increment of glucose quintile (Table 2).

**Table 1:** Baseline characteristics of study individuals in Me-Can.

Characteristics		Men	Women
**Baseline measurement, year**		1972–2005	1977–2005
**Individuals, ** ***n***		274,126	275,818
**Baseline age, y, mean (SD)**		44.7 (11.6)	45.0 (12.8)
Categories, *n* (%)	<30	24,756 (9)	30,461 (11)
	30 to <45	143,291 (52)	141,638 (51)
	45 to <60	73,567 (27)	65,793 (24)
	≥60	32,512 (12)	37,926 (14)
**Smoking status, ** ***n*** ** (%)**	Never smoker	110,154 (40)	137,767 (50)
	Ex-smoker	85,094 (31)	73,263 (27)
	Current smoker	77,995 (29)	64,097 (23)
	Missing	883 (0)	691 (0)
**BMI, kg/m^2^, mean (SD)**		25.8 (3.5)	25.0 (4.5)
Categories, *n* (%)	<25	120,026 (44)	159,700 (58)
	25 to <30	123,132 (45)	80,836 (29)
	≥30	30,968 (11)	35,282 (13)
**Follow-up, y, mean (SD)**		11.3 (7.4)	9.6 (4.4)
Categories, *n* (%)	<5	39,411 (14)	39,017 (14)
	5 to <15	184,479 (67)	206,769 (75)
	15 to <25	21,583 (8)	27,687 (10)
	≥25	28,653 (11)	2,345 (1)

**Table 2:** Characteristics of individuals within quintile levels of glucose.

Characteristics	Sex	Quintile 1–5				
		1	2	3	4	5
**Glucose, mmol/l, mean (SD)**	**Men**	4.1 (0.5)	4.7 (0.3)	5.1 (0.3)	5.5 (0.4)	6.9 (2.0)
	**Women**	4.0 (0.5)	4.6 (0.3)	4.9 (0.3)	5.3 (0.3)	6.5 (1.7)
**Baseline age, y, mean (SD)**	**Men**	42.5 (11.1)	43.3 (11.1)	44.1 (11.4)	45.1 (11.6)	48.1 (12.1)
	**Women**	41.9 (11.7)	43.0 (11.9)	44.3 (12.5)	45.7 (12.8)	49.5 (13.5)
**BMI, kg/m^2^, mean (SD)**	**Men**	25.2 (3.3)	25.5 (3.3)	25.7 (3.4)	26.0 (3.5)	26.7 (3.9)
	**Women**	24.0 (3.9)	24.5 (4.1)	24.8 (4.2)	25.2 (4.4)	26.4 (5.1)
**Current smoker, %**	**Men**	29	29	28	29	29
	**Women**	24	24	23	23	22

The mean follow-up time was 11.3 y (SD = 7.4) in men and 9.6 y (SD = 4.4) in women. Excluding the first year of observation, 18,621 men and 11,664 women were diagnosed with cancer during follow-up and 6,973 men and 3,088 women died of cancer.

### Glucose and RR of Cancer

Glucose was significantly positively associated with risk of overall incident and fatal cancer. In men, the RR (95% confidence interval [CI]) per 1 mmol/l increment was for incident cancer 1.05 (1.01–1.10), and for fatal cancer 1.15 (1.07–1.22) (Tables 3 and 4). In analysis of glucose in quintiles, the RR for the top versus bottom quintile was for incident cancer 1.18 (1.00–1.37, *p* for trend = 0.06), and for fatal cancer 1.50 (1.18–1.94, *p* for trend<0.001). Significant increases in risk of incident and fatal cancer at specific sites per 1 mmol/l increment in glucose among men were observed for cancer of the liver, gallbladder, and the respiratory tract. Significant linear associations were also found for incident thyroid cancer, multiple myeloma, and for fatal rectal cancer, and glucose in the top quintile was associated with a significant increased risk of fatal colon cancer.

**Table 3:** RR of incident cancer by glucose in quintiles and per 1 mmol/l increment.

Site (ICD-7)	Sex^b^	*n* Cases^c^	Quintile 1–5, RR (95% CI)^a^					*p* for trend	RR (95% CI) per 1 mmol/l increment^a^^,^ c
			1 (ref)	2	3	4	5		
**Total cancer**	**Men**								
	**Person-years**		550,091	545,386	517,011	588,557	537,656		
	***n*** ** cases**	**18,621**	3,346	3,437	3,265	4,234	4,339		
	**Rate** d		529	535	531	564	549		
	**RR**		1.00	1.07 (0.90–1.25)	1.10 (0.93–1.29)	1.18 (1.03–1.37)	1.18 (1.00–1.37)	0.06	1.05 (1.01–1.10)
	**Women**								
	**Person-years**		460,543	435,465	497,999	447,399	467,908		
	***n*** ** cases**	**11,664**	1,946	1,842	2,329	2,441	3,106		
	**Rate** d		383	367	376	409	424		
	**RR**		1.00	0.87 (0.70–1.07)	0.90 (0.73–1.10)	1.18 (0.97–1.42)	1.29 (1.07–1.59)	<0.001	1.11 (1.05–1.16)
**Lip, oral cavity, pharynx (140–149)**	**Men**	**453**	1.00	0.81 (0.29–2.34)	1.37 (0.48–3.86)	1.99 (0.76–5.31)	1.89 (0.70–5.10)	0.2	1.27 (0.97–1.66)
	**Women**	**128**	1.00	0.73 (0.08–6.46)	2.93 (0.44–19.6)	1.14 (0.15–8.65)	1.89 (0.28–13.0)	0.4	1.37 (0.87–2.14)
**Oesophagus (150)**	**All**	**246**	1.00	0.71 (0.18–2.89)	0.66 (0.16–2.70)	1.24 (0.35–4.55)	1.48 (0.41–5.33)	0.3	1.29 (0.92–1.80)
**Stomach (151)**	**Men**	**628**	1.00	0.68 (0.28–1.64)	1.07 (0.44–2.46)	0.76 (0.33–1.74)	0.81 (0.35–1.84)	0.5	0.93 (0.75–1.17)
	**Women**	**297**	1.00	0.84 (0.18–3.78)	2.34 (0.63–8.80)	1.84 (0.48–7.09)	2.65 (0.73–9.42)	0.2	1.31 (1.00–1.73)
**Colon (153)**	**Men**	**1,455**	1.00	0.93 (0.52–1.64)	0.97 (0.54–1.74)	0.73 (0.42–1.29)	1.33 (0.79–2.28)	0.2	1.02 (0.88–1.18)
	**Women**	**979**	1.00	0.97 (0.44–2.05)	1.03 (0.50–2.10)	1.03 (0.52–2.16)	1.33 (0.65–2.59)	0.5	0.99 (0.84–1.16)
**Rectum, anus (154)**	**Men**	**899**	1.00	1.74 (0.81–3.69)	1.94 (0.90–4.22)	2.52 (1.21–5.10)	1.69 (0.81–3.53)	0.5	1.14 (0.94–1.37)
	**Women**	**446**	1.00	0.84 (0.28–2.52)	0.79 (0.28–2.28)	1.18 (0.44–3.29)	1.00 (0.37–2.79)	0.7	1.09 (0.85–1.40)
**Liver, intrahepatic bile ducts (155.0)**	**Men**	**176**	1.00	0.84 (0.14–4.69)	1.74 (0.32–9.42)	0.37 (0.06–2.16)	3.45 (0.73–16.1)	0.02	1.76 (1.21–2.56)
	**Women**	**60**	1.00	0.02 (0.00–0.93)	0.35 (0.02–4.89)	0.73 (0.06–9.11)	0.52 (0.04–6.22)	0.7	1.70 (0.94–3.08)
**Gallbladder, biliary tract (155.1–155.3)**	**Men**	**79**	1.00	5.10 (0.37–70.2)	1.25 (0.06–23.6)	5.10 (0.38–67.0)	6.71 (0.52–86.4)	0.2	2.01 (1.14–3.53)
	**Women**	**77**	1.00	7.36 (0.40–133)	1.99 (0.11–38.1)	2.72 (0.14–49.8)	7.50 (0.52–110)	0.1	1.58 (0.96–2.61)
**Pancreas (157)**	**Men**	**418**	1.00	0.50 (0.16–1.55)	1.25 (0.42–3.78)	1.50 (0.54–4.22)	1.99 (0.73–5.53)	0.07	1.28 (0.97–1.68)
	**Women**	**230**	1.00	2.34 (0.40–13.6)	2.72 (0.52–14.1)	5.64 (1.14–28.4)	12.1 (2.65–55.1)	0.001	1.55 (1.12–2.13)
**Larynx, trachea/bronchus/lung (161, 162)**	**Men**	**2,294**	1.00	1.07 (0.68–1.69)	0.84 (0.52–1.33)	1.33 (0.87–2.10)	1.42 (0.90–2.16)	0.09	1.15 (1.02–1.29)
	**Women**	**659**	1.00	1.94 (0.81–4.69)	0.97 (0.40–2.28)	1.84 (0.79–4.22)	1.25 (0.54–2.93)	1.0	1.11 (0.89–1.38)
**Breast (170)**	**Women**	**4,094**	1.00	0.90 (0.65–1.29)	0.90 (0.65–1.25)	1.29 (0.93–1.79)	1.03 (0.73–1.42)	0.6	1.06 (0.98–1.16)
**Cervix uteri (171)**	**Women**	**280**	1.00	0.76 (0.21–2.65)	0.56 (0.16–1.89)	1.42 (0.42–4.59)	0.38 (0.11–1.42)	0.3	0.85 (0.59–1.21)
**Other parts of uterus (172, 174)**	**Women**	**762**	1.00	0.97 (0.40–2.40)	0.93 (0.40–2.22)	1.84 (0.79–4.13)	2.65 (1.21–5.86)	0.001	1.14 (0.95–1.38)^e^
**Endometrium (172)**	**Women**	**727**	1.00	1.03 (0.40–2.59)	0.90 (0.37–2.16)	1.89 (0.81–4.40)	2.59 (1.14–5.75)	0.003	1.14 (0.95–1.38)^e^
**Ovary (175.0)**	**Women**	**504**	1.00	0.52 (0.19–1.37)	0.76 (0.30–1.89)	0.50 (0.19–1.25)	0.58 (0.23–1.46)	0.3	0.85 (0.66–1.10)
**Prostate (177)**	**Men**	**5,713**	1.00	1.18 (0.87–1.59)	1.14 (0.84–1.55)	1.10 (0.84–1.46)	0.93 (0.70–1.21)	0.2	0.97 (0.90–1.04)
**Testis (178)**	**Men**	**220**	1.00	1.18 (0.32–4.31)	1.03 (0.28–3.95)	0.58 (0.14–2.40)	1.07 (0.25–4.59)	1.0	0.89 (0.59–1.34)
**Kidney, renal cell (180.0, 180.9)**	**Men**	**505**	1.00	3.07 (1.10–8.65)	3.45 (1.25–9.75)	2.28 (0.81–6.34)	2.65 (0.97–7.36)	0.4	1.14 (0.89–1.46)
	**Women**	**210**	1.00	0.37 (0.07–1.89)	0.60 (0.14–2.65)	0.52 (0.11–2.34)	0.81 (0.20–3.29)	0.8	1.02 (0.72–1.46)
**Bladder (181)**	**Men**	**1,280**	1.00	0.90 (0.48–1.64)	0.81 (0.42–1.50)	1.25 (0.70–2.22)	1.18 (0.65–2.16)	0.3	1.17 (1.00–1.37)
	**Women**	**227**	1.00	0.76 (0.14–4.31)	1.64 (0.35–7.63)	1.64 (0.35–7.77)	3.61 (0.87–15.4)	0.04	1.45 (1.05–2.01)
**Melanoma of skin (190)**	**Men**	**863**	1.00	1.37 (0.68–2.72)	1.00 (0.48–2.05)	0.90 (0.44–1.84)	0.87 (0.42–1.84)	0.7	0.92 (0.75–1.13)
	**Women**	**592**	1.00	0.73 (0.30–1.79)	0.60 (0.25–1.42)	0.63 (0.26–1.50)	1.14 (0.50–2.59)	0.5	1.04 (0.83–1.31)
**Nonmelanoma of skin (191)**	**Men**	**684**	1.00	0.35 (0.15–0.84)	0.97 (0.44–2.16)	0.65 (0.29–1.42)	0.56 (0.25–1.25)	0.6	0.96 (0.77–1.19)
	**Women**	**337**	1.00	1.21 (0.30–4.89)	1.89 (0.54–6.71)	1.55 (0.42–5.64)	3.07 (0.93–10.3)	0.05	1.17 (0.89–1.53)
**Brain, nervous tissue (193)**	**Men**	**331**	1.00	1.84 (0.60–5.64)	0.90 (0.28–2.93)	1.21 (0.40–3.78)	0.44 (0.13–1.50)	0.07	0.59 (0.42–0.84)
	**Women**	**201**	1.00	0.76 (0.14–3.95)	1.14 (0.24–5.20)	1.69 (0.37–7.63)	1.89 (0.42–8.35)	0.4	1.34 (0.92–1.94)
**Thyroid gland (194)**	**Men**	**97**	1.00	2.40 (0.24–25.1)	2.34 (0.21–25.4)	1.46 (0.14–16.1)	11.3 (1.29–98.3)	0.02	1.88 (1.16–3.07)
	**Women**	**180**	1.00	0.46 (0.10–2.10)	0.50 (0.12–2.10)	0.28 (0.06–1.29)	0.18 (0.04–0.87)	0.05	0.72 (0.47–1.10)
**Lymph/hematopoietic tissue (200–209)**	**Men**	**1,426**	1.00	1.07 (0.60–1.94)	0.68 (0.37–1.21)	1.50 (0.87–2.65)	1.25 (0.70–2.16)	0.3	1.10 (0.95–1.28)
	**Women**	**793**	1.00	0.70 (0.32–1.59)	0.76 (0.35–1.64)	0.73 (0.33–1.59)	1.18 (0.56–2.46)	0.3	1.19 (0.99–1.43)
**Non-Hodgkin's lymphoma (200, 202)**	**Men**	**634**	1.00	0.79 (0.33–1.79)	0.42 (0.17–1.00)	1.07 (0.50–2.40)	0.65 (0.28–1.50)	0.5	0.89 (0.71–1.13)
	**Women**	**378**	1.00	0.73 (0.23–2.40)	0.81 (0.28–2.52)	0.97 (0.32–3.00)	1.29 (0.44–3.78)	0.4	1.24 (0.95–1.61)
**Hodgkin's lymphoma (201)**	**All**	**113**	1.00	0.79 (0.11–5.64)	0.76 (0.11–5.54)	1.44 (0.23–9.36)	1.13 (0.16–7.93)	0.8	1.23 (0.73–2.06)
**Multiple myeloma (203)**	**Men**	**252**	1.00	1.14 (0.26–4.69)	0.87 (0.19–3.78)	1.79 (0.48–6.96)	2.93 (0.79–11.1)	0.04	1.59 (1.13–2.23)
	**Women**	**148**	1.00	0.32 (0.05–2.05)	0.48 (0.09–2.59)	0.12 (0.02–0.81)	0.84 (0.17–4.13)	0.7	0.92 (0.58–1.45)
**Leukemia (204–207)**	**Men**	**398**	1.00	2.34 (0.76–7.23)	1.37 (0.42–4.59)	2.93 (0.97–8.65)	2.22 (0.73–6.71)	0.2	1.17 (0.89–1.54)
	**Women**	**192**	1.00	0.42 (0.08–2.28)	0.81 (0.17–3.78)	0.56 (0.11–2.72)	0.84 (0.19–3.78)	0.8	1.29 (0.90–1.86)
**Other cancer** f	**Men**	**909**	1.00	0.87 (0.42–1.79)	1.50 (0.73–3.14)	1.59 (0.79–3.22)	1.46 (0.70–3.00)	0.4	1.12 (0.92–1.36)
	**Women**	**583**	1.00	1.03 (0.38–2.79)	0.60 (0.23–1.55)	1.99 (0.81–4.99)	2.52 (1.03–6.10)	0.006	1.33 (1.07–1.65)

a RRs reported with three significant figures, estimated from Cox models with attained age as time scale, stratified by cohort, sex, and birth year, and adjusted for baseline age, BMI, and smoking status, and RRs per 1 mmol/l were additionally adjusted for fasting time. RRs are corrected for RDR; conversion into uncorrected RR = exp(log(RR)×RDR). RDR quintiles: men, 0.30; women, 0.30; all, 0.31. RDR per 1 mmol/l: men, 0.40; women, 0.43; all, 0.41.

b RRs are presented separately for men and women if the number of cases in each group was >50, and combined if the number of cases in each group was ≤50 and if the total number of cases >80.

c RRs per 1 mmol/l increment included individuals with glucose levels <10 mmol/l (99% of individuals). Number of cases corresponds to quintile analyses, which included all individuals.

d Per 100,000 person-years, age-standardized to the European standard population.

e RRs were significant in analyses that also included individuals with glucose levels ≥10 mmol/l.

f Other cancer than the separately presented sites.

ICD-7, International Classification of Diseases, seventh revision; ref, referent group.

**Table 4:** RR of overall death and of fatal cancer by glucose in quintiles and per 1 mmol/l increment.

Site (ICD-7)	Sex^b^	*n* cases^c^	Quintile 1–5, RR (95% CI)^a^					*p* for trend	RR (95% CI) per 1 mmol/l increment^a^^,^ c
			1 (ref)	2	3	4	5		
**Overall death**	**Men**								
	**Person-years** d		510,654	508,473	477,979	545,596	496,955		
	***n*** ** cases**	**21,445**	3,721	3,644	3,523	4,552	6,005		
	**Rate** e		766	752	745	780	932		
	**RR**		1.00	0.90 (0.79–1.07)	1.07 (0.90–1.25)	1.07 (0.90–1.21)	2.22 (1.94–2.52)	<0.001	1.29 (1.24–1.33)
	**Women**								
	**Person-years** d		423,829	398,725	458,898	409,746	428,549		
	***n*** ** cases**	**8,424**	1,142	1,074	1,455	1,644	3,109		
	**Rate** e		355	320	338	366	463		
	**RR**		1.00	0.73 (0.54–0.97)	0.76 (0.58–0.97)	1.03 (0.81–1.33)	2.34 (1.84–2.93)	<0.001	1.36 (1.29–1.43)
**Total cancer**	**Men**								
	***n*** ** cases**	**6,973**	1,271	1,223	1,191	1,549	1,739		
	**Rate** e		238	221	228	236	246		
	**RR**		1.00	0.90 (0.68–1.18)	1.10 (0.84–1.42)	1.14 (0.87–1.46)	1.50 (1.18–1.94)	<0.001	1.15 (1.07–1.22)
	**Women**								
	***n*** ** cases**	**3,088**	472	430	581	653	952		
	**Rate** e		118	108	119	128	139		
	**RR**		1.00	0.79 (0.50–1.21)	0.84 (0.56–1.25)	1.29 (0.87–1.94)	1.69 (1.18–2.52)	<0.001	1.21 (1.11–1.33)
**Lip, oral cavity, pharynx (140–149)**	**All**	**180**	1.00	1.57 (0.30–8.34)	1.07 (0.18–6.08)	3.32 (0.69–16.0)	5.33 (1.13–24.9)	0.01	1.50 (1.00–2.25)
**Oesophagus (150)**	**All**	**187**	1.00	0.61 (0.11–3.39)	0.69 (0.12–3.86)	2.45 (0.55–10.8)	4.65 (1.10–20.2)	0.005	1.73 (1.19–2.53)
**Stomach (151)**	**Men**	**438**	1.00	0.63 (0.23–1.74)	0.68 (0.24–1.89)	0.65 (0.25–1.79)	0.60 (0.23–1.59)	0.3	0.94 (0.72–1.23)
	**Women**	**198**	1.00	1.07 (0.16–6.71)	1.84 (0.35–9.91)	1.50 (0.26–8.35)	5.31 (1.10–25.4)	0.01	1.56 (1.13–2.14)
**Colon (153)**	**Men**	**567**	1.00	0.81 (0.30–2.16)	1.18 (0.46–3.07)	1.07 (0.42–2.65)	2.72 (1.14–6.46)	0.004	1.09 (0.87–1.37)^f^
	**Women**	**306**	1.00	0.38 (0.10–1.55)	0.38 (0.11–1.37)	0.40 (0.11–1.42)	0.97 (0.30–3.07)	0.4	1.15 (0.87–1.52)
**Rectum, anus (154)**	**Men**	**332**	1.00	0.84 (0.23–3.14)	1.21 (0.33–4.50)	2.86 (0.90–9.26)	2.93 (0.90–9.58)	0.02	1.44 (1.08–1.92)
	**Women**	**125**	1.00	0.20 (0.02–2.22)	1.33 (0.19–9.42)	1.79 (0.26–12.2)	1.03 (0.15–6.96)	0.6	1.11 (0.71–1.74)
**Liver, intrahepatic bile ducts (155.0)**	**All**	**134**	1.00	0.36 (0.05–2.63)	0.64 (0.10–4.28)	0.38 (0.06–2.45)	2.22 (0.43–11.5)	0.05	1.77 (1.19–2.62)
**Pancreas (157)**	**Men**	**450**	1.00	0.65 (0.23–1.89)	1.00 (0.33–2.86)	0.84 (0.30–2.34)	2.34 (0.90–6.22)	0.02	1.24 (0.95–1.61)^f^
	**Women**	**262**	1.00	2.34 (0.42–12.8)	2.22 (0.44–10.9)	4.79 (1.00–22.4)	12.8 (3.00–54.6)	<0.001	1.70 (1.29–2.24)
**Larynx, trachea/bronchus/lung (161, 162)**	**Men**	**1,846**	1.00	0.93 (0.56–1.55)	1.03 (0.60–1.74)	1.59 (0.97–2.59)	1.59 (0.97–2.59)	0.03	1.21 (1.06–1.37)
	**Women**	**433**	1.00	1.37 (0.44–4.22)	0.76 (0.25–2.28)	2.52 (0.90–7.23)	1.89 (0.68–5.31)	0.2	1.29 (1.00–1.65)
**Breast (170)**	**Women**	**387**	1.00	0.87 (0.28–2.79)	1.14 (0.40–3.37)	1.25 (0.42–3.69)	0.87 (0.30–2.59)	0.7	0.97 (0.74–1.28)
**Cervix uteri (171)**	**Women**	**51**	1.00	6.10 (0.14–253)	3.00 (0.07–125)	32.8 (1.10–994)	21.2 (0.68–662)	0.04	2.26 (1.20–4.28)
**Other parts of uterus (172, 174)**	**Women**	**81**	1.00	0.33 (0.01–9.11)	0.70 (0.04–12.8)	0.84 (0.05–14.7)	9.26 (0.79–109)	0.003	1.69 (1.05–2.73)
**Ovary (175.0)**	**Women**	**249**	1.00	0.14 (0.03–0.63)	0.44 (0.12–1.59)	0.30 (0.08–1.18)	0.50 (0.14–1.74)	0.9	0.94 (0.67–1.32)
**Prostate (177)**	**Men**	**817**	1.00	1.29 (0.63–2.79)	1.29 (0.60–2.79)	0.65 (0.30–1.42)	0.81 (0.38–1.69)	0.4	0.97 (0.80–1.18)
**Kidney, renal cell (180.0, 180.9)**	**Men**	**197**	1.00	1.50 (0.30–7.50)	2.46 (0.52–11.9)	0.76 (0.15–3.86)	1.79 (0.38–8.20)	0.8	1.25 (0.84–1.87)
	**Women**	**59**	1.00	0.73 (0.04–13.9)	0.25 (0.01–4.69)	0.02 (0.00–0.73)	0.87 (0.07–11.1)	0.6	0.94 (0.48–1.85)
**Bladder (181)**	**All**	**250**	1.00	0.26 (0.07–1.00)	0.59 (0.16–2.22)	0.43 (0.12–1.57)	0.82 (0.24–2.76)	0.6	1.10 (0.77–1.55)
**Melanoma of skin (190)**	**All**	**220**	1.00	3.32 (0.71–15.3)	3.54 (0.76–16.6)	4.28 (0.97–19.0)	4.20 (0.94–18.7)	0.2	1.10 (0.74–1.63)
**Lymph/hematopoietic tissue (200–209)**	**Men**	**611**	1.00	0.60 (0.26–1.46)	0.44 (0.17–1.07)	1.00 (0.44–2.22)	0.81 (0.35–1.84)	0.9	1.06 (0.84–1.34)
	**Women**	**237**	1.00	1.29 (0.30–5.53)	0.48 (0.11–1.99)	0.56 (0.14–2.34)	0.65 (0.17–2.52)	0.6	0.90 (0.64–1.26)
**Other cancer** g	**Men**	**929**	1.00	1.10 (0.54–2.28)	1.84 (0.90–3.78)	0.90 (0.46–1.84)	1.18 (0.58–2.40)	0.9	1.01 (0.83–1.22)
	**Women**	**513**	1.00	0.93 (0.30–2.79)	1.14 (0.42–3.22)	2.59 (0.97–6.96)	1.94 (0.73–5.10)	0.1	1.24 (0.99–1.54)

a RRs reported with three significant figures, estimated from Cox models with attained age as time scale, stratified by cohort, sex, and birth year, and adjusted for baseline age, BMI, and smoking status, and RRs per 1 mmol/l were additionally adjusted for fasting time. RRs are corrected for RDR; conversion into uncorrected RR = exp(log(RR)×RDR). RDR quintiles: men, 0.30; women, 0.30; all, 0.31. RDR per 1 mmol/l: men, 0.40; women, 0.43; all, 0.41.

b RRs are presented separately for men and women if the number of cases in each group was >50, and combined if the number of cases in each group was ≤50 and if the total number of cases was >80.

c RRs per 1 mmol/l increment included individuals with glucose levels <10 mmol/l (99% of individuals). Number of cases corresponds to quintile analyses, which included all individuals.

d Person-years for cancer death corresponds to those for overall death.

e Per 100,000 person-years, age-standardized to the European standard population.

f RRs were significant in analyses that also included individuals with glucose levels ≥10 mmol/l.

g Other cancer than the separately presented sites.

ICD-7, International Classification of Diseases, seventh revision; ref, referent group.

In women, the association between a 1 mmol/l increase in glucose level and overall cancer was somewhat stronger than in men; the RR among women for incident cancer was 1.11 (1.05–1.16), and for fatal cancer 1.21 (1.11–1.33) (Tables 3 and 4). Significant positive associations among women were observed for incident and fatal cancer of the pancreas, and stomach (borderline significant for incidence). A significant linear association was also observed for incident urinary bladder cancer and for fatal cervix and uterine corpus cancer. Furthermore, top quintile level of glucose was significantly associated with an increased risk of incident endometrial cancer, and a decreased risk of incident thyroid cancer.

In men and women combined, a 1 mmol/l increment in glucose level was associated with an increased risk of death from cancer of the oropharynx and oesophagus.

BMI and fasting time before blood draw had no effect on the association between glucose and risk of cancer overall in men or in women (*p* for interaction, all >0.05). There was no significant interaction between glucose and subcohort on the risk of incident and fatal cancer in men, or for fatal cancer in women (*p* for interaction, all >0.05). However, the association between glucose and risk of incident cancer in women differed significantly between the cohorts; the overall *p*-value for interaction was 0.02, and the RR per 1 mmol/l increment of glucose ranged between 0.98 (0.84–1.12) in the 40-y cohort, and 1.30 (1.15–1.50) in the VIP. No similar pattern was observed in men, among whom the RR for incident cancer was lowest in the VIP (RR = 0.95) and highest in the VHM&PP.

### Decile Levels of Glucose and Risk

**Figure 2:**
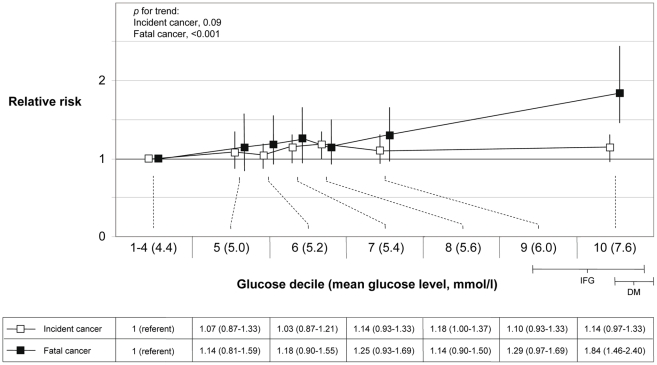
RR (95% CI) in men of incident (*n* = 18,621) and fatal (*n* = 6,973) cancer by deciles of glucose. The risk estimates for decile categories are plotted on the *x*-axis at the mean glucose level for each decile category. IFG indicates the range of impaired fasting glucose in the cohorts among individuals that had fasted more than 8 h before the blood draw, and DM indicates the range of diabetic glucose levels. Glucose levels in the Oslo study I were recalculated (level −0.95) to correspond with enzymatic levels.

**Figure 3:**
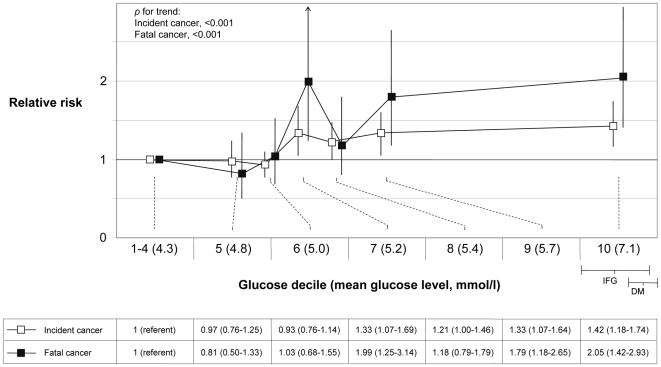
RR (95% CI) in women of incident (*n* = 11,664) and fatal (*n* = 3,088) cancer by deciles of glucose. The risk estimates for decile categories are plotted on the *x*-axis at the mean glucose level for each decile category. IFG indicates the range of impaired fasting glucose in the cohorts among individuals that had fasted more than 8 h before the blood draw, and DM indicates the range of diabetic glucose levels.

We further explored risk of cancer by decile categories of glucose levels. In order to use a broad referent category that includes healthy normal glucose levels, we used the lowest 40% of glucose levels as referent group. Among fasting individuals, the cut-off for impaired fasting glucose was in the top 10%–20% of glucose levels. The association between glucose level and cancer risk was approximately linear across the full range of glucose levels (Figures 2 and 3), and the extension of a linear model with an x^2^ variable did not significantly improve the fit of the association with incident or fatal cancer among men or women (*p*, all >0.05). In men, the RR for top decile versus decile 1–4 for incident cancer was 1.14 (0.97–1.33, *p* for trend = 0.09), and for fatal cancer 1.84 (1.46–2.40, *p* for trend<0.001). RRs of total cancer, excluding prostate cancer, were for incident cancer 1.37 (1.14–1.64, *p* for trend = 0.002), and for fatal cancer 2.10 (1.59–2.72, *p* for trend<0.001). In women, the RR for top decile versus decile 1–4 for overall incident cancer was 1.42 (1.18–1.74, *p* for trend<0.001), and for fatal cancer 2.05 (1.42–2.93, *p* for trend<0.001). The corresponding RR for overall death was in men 3.29 (2.86–3.78, *p* for trend<0.001), and in women 3.69 (3.00–4.59, *p* for trend<0.001).

The absolute risk of incident cancer over a 20-y period for a 50-y old man in decile 1–4 and decile 10 of glucose was 14.0% and 15.7%, respectively, and the corresponding risk of fatal cancer was 5.0% and 8.8%. In women, the corresponding absolute risks of developing cancer were 12.2% and 16.7%, and for cancer death, 3.0% and 6.0%, respectively.

## Discussion

In this large prospective cohort study, elevated blood glucose was significantly associated with an increased risk of incident and fatal cancer at all sites combined, and of several specific cancers. In women, a linear association between glucose and risk of overall incident and fatal cancer was observed, and levels within the upper normal range were also related to increases in risk. In men, the association between glucose and total incident cancer was somewhat weaker, and risk of fatal cancer was only significantly increased at levels approximately equivalent to impaired glucose levels. Women in the top glucose decile had twice the risk of fatal cancer compared to women with glucose levels below the 40th percentile and the risk increase among men in the top decile was almost the same. Risk estimates were obtained after correction for random error in glucose levels, which was high in our study in accordance with previous observations [3],[8],[9]. The estimates of excess risk of fatal cancer in the top decile corrected for regression dilution were 4-fold higher than the uncorrected estimates. These data indicate that in previous analyses without such correction, risk estimates for increasing glucose may have been underestimated [1]–[7].

Results from our study and those from the largest study reported to date, on men and women in Korea [1], were largely congruent and together these studies provide strong evidence that high blood glucose is a risk factor for cancer. In our study, associations between glucose and overall incident and fatal cancer were stronger in women than in men, whereas in the Korean study, stronger associations were reported for men, for whom a significant increased risk of fatal cancer was observed already at levels below impaired fasting glucose. These differences between studies may be explained by different proportions of specific cancers in the populations. For example, prostate cancer is much more common in Europe than in Asia [30], and as glucose was not related to prostate cancer in either study, exclusion of prostate cancer in analyses of total cancer in our study strengthened the association with cancer. Type 2 diabetes has consistently been related to an increased risk of cancer at many sites [1],[31]–[33], and the findings in our and the Korean study suggest also that impaired fasting glucose levels, and to a lesser extent, also glucose levels within the upper normal range are associated with an increased risk of cancer.

Specific cancers for which there were strong associations between glucose and risk of incident and fatal cancer in the Korean study [1] and in our study, were pancreatic cancer, particularly in women, and liver cancer in men. Moreover, both studies showed strong associations between elevated glucose and risk of fatal cancer of the oesophagus and cervix uteri, and of fatal colorectal cancer in men. In our study, elevated glucose was also associated with an increased risk of cancer of the respiratory tract in men, and of gastric cancer in women, whereas no such associations were found in the Korean study. Smoking is strongly related to lung cancer and gastric cancer [34], and confounding or interaction between glucose and smoking may possibly explain the divergent findings. The proportion of current smokers in men was 29% in our study and 59% in the Korean study, and corresponding proportions were 23% and 4% in women. We observed no confounding or effect modification by smoking status in analyses of these cancers, but residual confounding may be present owing to an imprecise or incorrect categorisation of smoking status.

Our study is the first to report data on glucose and risk of oropharyngeal cancer, and suggests an increased risk of death from these cancers in individuals with elevated glucose. Furthermore, data on prediagnostic glucose levels and risk of multiple myeloma and thyroid cancer have previously been reported only from the VHM&PP cohort [2]. We found a significant increase in risk of these cancers in men with high glucose, whereas intriguingly, risk of thyroid cancer was markedly decreased in women with high glucose. Incidence rates of thyroid cancer are 2–3 times higher in women than in men, possibly influenced by female sex hormones [35]–[37], and we speculate that an interaction between sex hormones and glucose may underlie our findings, alternatively the results may be a chance finding.

Insulin and bioavailable insulin-like growth factor-I (IGF-I) are possible links between glucose and cancer; hyperglycaemia induces elevation of these hormones that stimulate tumour growth [38]. Glucose may also have a direct tumour-promoting effect as glucose is used as an energy substrate in tumour cells, particularly in fast-growing, highly proliferative tumour cells [39]–[41]. However, the importance of extracellular glucose concentration for tumour growth—and thereby a direct link between glucose itself and cancer risk—is unclear.

Although the link between glucose and cancer may be causal, confounding may also be involved. We controlled for two major putative confounders, BMI and smoking, and found that the association between glucose and cancer risk remained after adjustment for these factors. However, other putative confounding factors may be relevant. For example, a genetic variant with opposite effects on risk of type 2 diabetes and prostate cancer has recently been reported [42], and this could partly explain the null association between glucose and prostate cancer in our study as well as the consistently reported reduced risk of prostate cancer in men with type 2 diabetes [43]. Various lifestyle factors, related to glucose but with other pathways to cancer, are also potential confounders, e.g., alcohol for cancer of the oropharynx, oesophagus, liver, and colorectum, salt for gastric cancer, and physical activity and fruit and vegetable consumption for a number of cancers [44].

The association between glucose and cancer risk was stronger for fatal cancer overall and at several sites than for incident cancer. The explanation for this difference may vary between cancer types. Possibly, high glucose and related factors are more important for tumour progression than for tumour initiation. Alternatively, persons with high glucose may be diagnosed with cancer at a later stage, e.g., because of different health care seeking behaviour, or the results may be caused by inconsistencies in classification of cancer diagnosis versus cause of death [45],[46].

Previous studies have consistently shown an association between elevated glucose levels and risk of cardiovascular disease and also to all cause mortality [1],[47]–[49]. Accordingly, we found that elevated glucose was strongly related to an increased risk of all cause mortality; glucose levels in the top decile were related to a more than 3-fold increased risk. Our data indicate that glucose control by a healthy diet and physical activity may decrease risk of cancer at many sites in addition to a decreased risk of cardiovascular disease.

Strengths of our study include the large sample size from six European population-based cohorts with virtually complete capture of cancer cases [2],[50],[51], the use of incident as well as fatal cancer as endpoints, and the correction of risk estimates for intra-individual variation of glucose levels based on a large number of repeated measurements. In all cohorts, data were available for BMI and smoking status, and these factors were used as adjustment in analyses. Limitations of our study include the lack of data on other covariates that may have influenced risk estimates, and the different protocols for measurement of glucose applied in subcohorts, which invalidated the use of absolute glucose levels to our data.

In conclusion, abnormal glucose metabolism, independent of BMI, is associated with increases in risk of cancer and cancer death overall and at many specific sites. Furthermore, our data showed a linear and somewhat stronger association among women than among men, and the association was stronger for fatal compared to incident cancer.

## Abbreviations

40-y: Age 40-programme
BMI: body mass index
CI: confidence interval
CONOR: Cohort of Norway
Me-Can: Metabolic syndrome and Cancer project
MPP: Malmö Preventive Project
NCS: Norwegian Counties Study
Oslo: Oslo study I
RDR: regression dilution ratio
RR: relative risk
SD: standard deviation
VHM&PP: Vorarlberg Health Monitoring and Prevention Programme
VIP: Västerbotten Intervention Project
